# A study on the differences in urinary iodine and serum iodine in relation to children’s iodine nutrition and lipids

**DOI:** 10.1186/s12944-026-02982-7

**Published:** 2026-06-04

**Authors:** Yan Zhang, Lili Xu, Lingbo Wang, Haiyan Gao, Jiamin Hao, Le Dong, Lili Qiu, Jiahui Wang, Shichun Yan, Lixiang Liu

**Affiliations:** 1 Centre for Endemic Disease Control, Chinese Centre for Disease Control and Prevention, Harbin Medical University, Harbin City, Heilongjiang Province 150081 People’s Republic of China; 2 Key Laboratory of Etiology and Epidemiology, National Health Commission & Education Bureau of Heilongjiang Province, Harbin Medical University, Harbin City, Heilongjiang Province 150081 People’s Republic of China; 3 Heilongjiang Provincial Key Laboratory of Trace Elements and Human Health, Harbin Medical University, Harbin City, Heilongjiang Province 150081 People’s Republic of China; 4 Heilongjiang Provincial Center for Disease Control and Prevention, Harbin City, Heilongjiang Province 150081 People’s Republic of China; 5 Infection Control Department of The Fourth Affiliated Hospital of Harbin Medical University, Harbin City, 150081 Heilongjiang Province People’s Republic of China; 6 The Sixth Affiated Hospital of Harbin Medical University, Harbin City, Heilongjiang Province 150081 People’s Republic of China; 7 Endocrinology Department of the Second Affiliated Hospital of Harbin, Medical University, Harbin City, Heilongjiang Province 150081 People’s Republic of China

**Keywords:** Adolescents, Children, Dyslipidemia, Iodine

## Abstract

The safe intake range for iodine is narrow, and abnormal nutritional status (deficiency or excess) may affect children’s lipid metabolism by interfering with thyroid function. Following the widespread implementation of iodized salt in China, urinary iodine levels in children in some regions have exceeded the appropriate range. Nonetheless, the connection between iodine nutritional status and childhood dyslipidemia remains unclear. This study examined the associations between iodine status and lipid profiles among 6–17-year-old children and adolescents residing in regions of China where iodized salt is supplemented. The correlation between urinary iodine concentration (UIC), serum iodine concentration (SIC), and total cholesterol (TC) and triglyceride (TG) was explored using kernel density plots and chi-square tests. The logistic regression model incorporated covariates (age, sex, BMI) to control for potential confounding factors and examined the relationship between UIC, SIC, and lipid profile; meanwhile, stratified analysis and interaction analysis were conducted to test the effect modification of these variables on the aforementioned associations. Restricted cubic splines models based on logistic regression were utilized to examine dose-response relationships involving UIC, SIC and dyslipidemia. The results revealed that hypertriglyceridemia followed a U-shaped pattern across UIC groups, whereas hypercholesterolemia rose steadily with increasing UIC. Both outcomes showed U-shaped associations with SIC. After adjustment, UIC ≥ 300 µg/L was linked with elevated TC [OR 1.455 (1.175–1.802)]. Elevated TG was also more likely when the SIC was < 63.50 µg/L [OR 1.305(1.022,1.665)] or ≥ 82.28 µg/L [1.536(1.204,1.960)]. SIC is in a U-relationship with TG levels, whereas SIC values > 74.66 µg/L appeared to interact synergistically with TG. In conclusion, high urinary iodine is a risk factor for hypercholesterolemia, but this association is modified by BMI. Both low serum iodine and high serum iodine are linked to a higher likelihood of hypertriglyceridemia, exhibiting a non-linear relationship.

**Graphical Abstract:**

**Figure:**
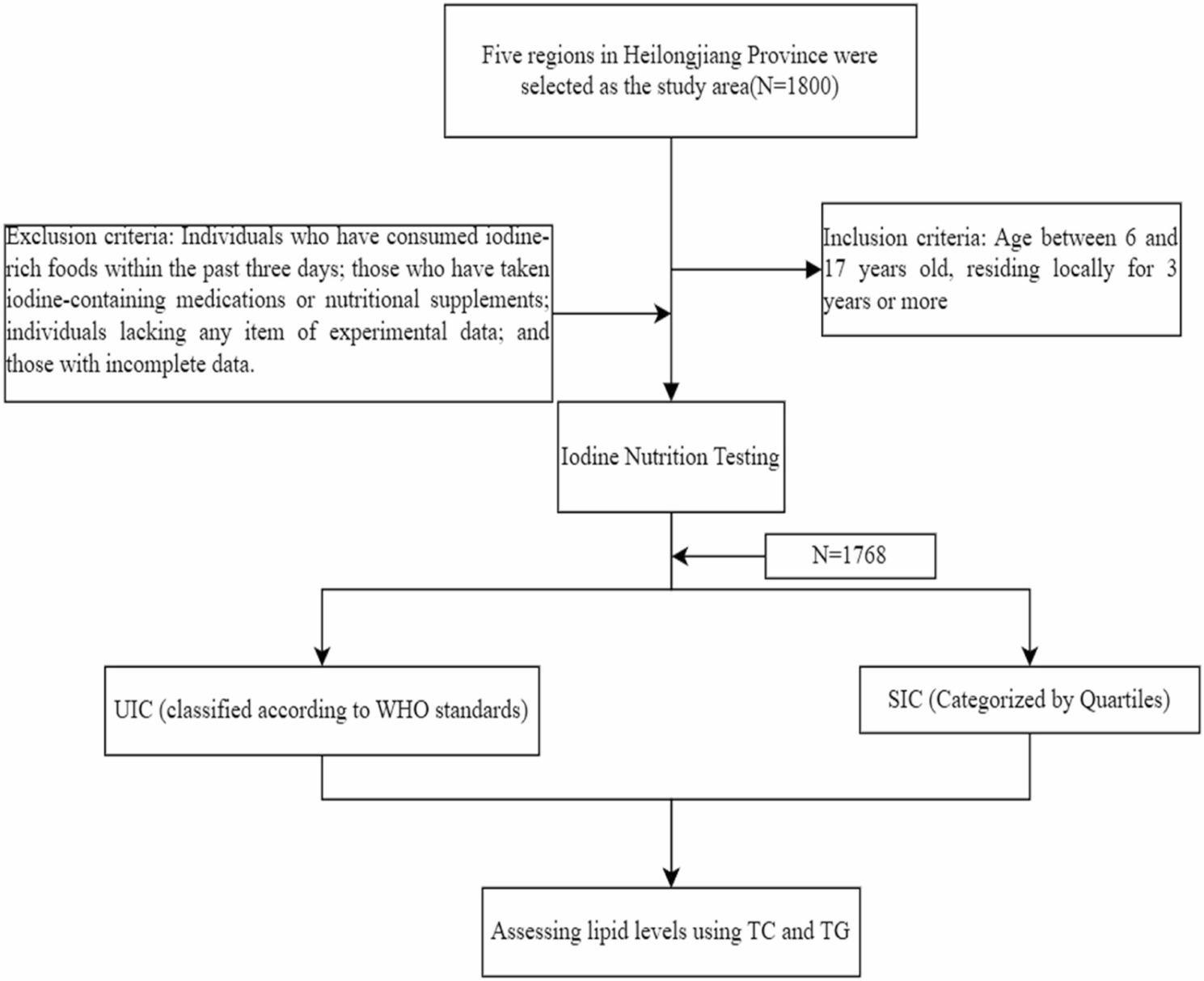

**Supplementary Information:**

The online version contains supplementary material available at 10.1186/s12944-026-02982-7.

## Introduction

Iodine is a fundamental micronutrient for metabolism, growth, and development, serving as the primary raw material for the synthesis of thyroxine in the human body. Thyroid problems can be brought on by both excessive and insufficient iodine consumption, which can result in metabolic problems. In the general population, iodine intake originates primarily from the environment, drinking water, and diet [1]. Iodine deficiency disorders (IDDs) represent a complex group of endemic diseases caused by insufficient iodine intake due to natural environmental iodine scarcity. These disorders are widely distributed across multiple provinces and regions in China and predominantly occur in environmentally iodine-deficient areas [2]. Over the past few decades, the implementation of the nationwide salt iodization policy has significantly improved iodine nutrition [3]. However, increased iodine consumption has resulted from the growing popularity of iodized salt programs [4]. Although the median urinary iodine level in children aged 8 to 10 years nationwide was 212.40 µg/L in 2022 [5], there is still a possibility of iodine excess, according to recent national iodine deficiency illness surveillance data. In Chinese and Korean populations, recent research has shown a link between iodine status and metabolic illnesses such as metabolic syndrome, hypertension, hyperglycemia, central obesity, dyslipidemia, and low HDL-related components [6].

Dyslipidemia typically refers to elevated levels of cholesterol and/or triglycerides (TGs) in serum, commonly known as hyperlipidemia. In recent years, owing to changes in lifestyle, dietary habits, and genetic predispositions, the prevalence of dyslipidemia among children has significantly increased both domestically and internationally [7]. This growing trend is concerning because the risk of childhood dyslipidemia persists into adulthood and can predict both fatal and non-fatal cardiovascular events, posing a public health challenge that impacts health and societal development [8]. Research has revealed a connection between thyroid function status and lipid metabolism, since thyroid dysfunction affects normal lipid metabolism in the body [9, 10]. Iodine affects thyroid function either directly or indirectly because it is a necessary trace element for the synthesis of thyroid function. Consequently, iodine nutrition may regulate lipid metabolism by affecting thyroid function [11].

Iodine shortage in adults can result in subclinical hypothyroidism, which raises the risk of subclinical inflammation, insulin resistance, and dyslipidemia [12]. Studies have shown that the prevalence of hypercholesterolemia and hypertriglyceridemia in adults has a U-shaped relationship with the urinary iodine concentration (UIC) and an inverted U-shaped relationship with the water iodine concentration (WIC) [13, 14]. Animal studies also indicate that excessive potassium iodate (KIO_3_) significantly elevates serum TG and TC in rats, with TG and TC abnormalities appearing earlier, suggesting that blood lipids may be the most sensitive indicator of high iodine exposure [15, 16]. These findings are mostly based on evaluations of iodine nutritional indicators in adult populations, despite the fact that several research studies have shown links between iodine excess and deficiency and blood lipid levels. The relationship between individual iodine nutritional status and blood lipids remains unclear, and research on the association between children’s iodine nutritional status and blood lipids remains scarce.

This study included children and adolescents aged 6–17 as research participants. For the first time, it simultaneously uses UIC and SIC as two indicators to evaluate children’s iodine nutritional status, revealing the relationship between iodine and blood lipids from the perspectives of intake and in vivo metabolism. This study aims to explore the potential effects of different iodine nutritional statuses on children’s lipid metabolism, in order to provide a scientific basis for the development of targeted iodine intervention programs and the early detection and management of dyslipidemia.

## Materials and methods

### Participants and survey regions

This study employed a transverse survey design with a multistage stratified cluster sampling method. Five regions in Heilongjiang Province—Huanan, Fuyu, Dongning, Zhaozhou, and Muling—were selected as study areas. These areas included both iodine-deficient and iodine-supplemented regions, characterized by median water iodine concentrations below 10 µg/L and iodized salt coverage of at least 95%. Within each selected region, two primary schools and two junior high schools were randomly selected for participation. A stratified random sampling method was applied at the grade level, with approximately 20 students randomly selected from each grade between grades 1 and 9, ensuring equal representation of male and female students A total of 1,768 children and adolescents participated in this study. The following were the criteria for inclusion and exclusion: (1) age 6–17 years with ≥ 3 years of local residence. (2) No family history of autoimmune diseases, endocrine abnormalities, thyroid disease, or genetic issues. (3) No consumption of iodine-rich foods within the past three days. (4) No intake of iodine-containing medications or supplements. (5) No missing laboratory data. (6) Participants with incomplete data. Written informed consent was obtained from parents or legal guardians, and assent was also obtained from all participating children. All collected personal data were coded during system entry to protect participant privacy. In any future reporting of study findings, all data will be presented in aggregated form to ensure that no identifiable personal information is disclosed.

### Survey techniques

To gather demographic data, such as sociodemographic and health status traits, a survey questionnaire was created. Age, body mass index (BMI), and sex (male or female) were the variables that were included of the statistical analysis model. The selection of covariates was based on prior literature [17, 18]. Members of the steering committee oversaw the administration of the questionnaire by qualified personnel who had completed performance reviews.

### Clinical diagnosis and laboratory testing

Urinary iodine and serum iodine levels were measured using the As³⁺-Ce⁴⁺ catalytic spectrophotometric method recommended by the National Reference Laboratory for Iodine Deficiency Disorders (NRLIDD) and the Chinese Center for Disease Control and Prevention (CDC). Lipid abnormalities were assessed using total cholesterol (TC) and triglyceride (TG) levels, which were identified with a Roche P800 fully automated biochemical analyzer. Triglycerides were measured using the phosphoglycerate oxidase-4-chlorate method. Thyroid hormones (FT3: free triiodothyronine. FT4: free thyroxine. TSH: thyroid-stimulating hormone) were determined via chemiluminescent immunoassay, following the kit instructions.

The participants fasted overnight, and in the morning, blood and urine samples were taken. Blood samples were centrifuged to obtain serum supernatants, and both the urine and blood samples were stored at − 80 °C until analysis. TC and TG were measured using a Roche P800 fully automated biochemical analyzer and served as the primary indicators of dyslipidemia. Lipid classifications followed the 2023 Chinese Lipid Management Guidelines. For TC: borderline high was defined as 4.40–5.20 mmol/L, and hypercholesterolemia as ≥ 5.20 mmol/L. For TG: among children ≤ 10 years, borderline high was defined as 0.80–1.10 mmol/L and hypertriglyceridemia as ≥ 1.10 mmol/L; among those ≥ 10 years, borderline high was defined as 1.00–1.50 mmol/L and hypertriglyceridemia as ≥ 1.50 mmol/L (Jj et al., 2023). Thyroid hormone reference ranges were as follows: FT4, 7.98–29.6 pmol/L; FT3, 2.50–9.00 pmol/L for ages 2–8 years and 3.00–8.55 pmol/L for ages 9–18 years; TSH, 0.4–6.2 mIU/L for ages 2–8 years and 0.4–5.36 mIU/L for ages 9–18 years. Serum iodine concentrations were categorized into quartiles: < 63.50 µg/L, 63.50–72.62 µg/L, 72.62–82.28 µg/L, and ≥ 82.28 µg/L. Urinary iodine status was classified according to the Guidelines for Monitoring the Salt Iodization Program and Evaluating Population Iodine Status, in which a median UIC < 100 µg/L indicates iodine deficiency, 100–299 µg/L indicates adequate iodine, and ≥ 300 µg/L indicates iodine excess. BMI: Determined by dividing weight (kg) by height² (m2). BMI is divided into four categories: underweight, normal, overweight, and obese, in accordance with the national health industry standards “Screening for Malnutrition in School-Age Children and Adolescents” (WS/T 456–2014) and “Screening for Overweight and Obesity in School-Age Children and Adolescents” (WS/T 586–2018).

### Statistical analysis

Data processing and statistical analysis were conducted using R 4.5.0. The normality of continuous variables was assessed using the Shapiro-Wilk test in combination with Q-Q plots and histograms. Lipid levels are expressed as medians [M (P25, P75)], and intergroup comparisons were conducted using the Mann-Whitney U test and the Kruskal-Wallis H test. It should be noted that in the stratified analysis, serum iodine (SIC) was divided into three groups according to quartiles to facilitate result presentation and clinical interpretation. It is important to emphasize that these cutoff values are primarily used for subgroup analysis rather than defining clinical diagnostic criteria. Kernel density plots and chi-square tests were used to explore associations between varying iodine nutritional statuses and dyslipidemia. Thyroid function levels and lipid levels were examined using Spearman correlation analysis. To address potential confounders, a series of multivariable logistic regression models were utilized, including an unadjusted model (Model 1) and a model adjusted for age, sex, and BMI (Model 2). These models were used to estimate odds ratios (ORs) and corresponding 95% confidence intervals (CI) to identify factors linked to dyslipidemia in relation to urinary and serum iodine concentrations. Product interaction terms between exposure variables (SIC, UIC) and continuous variables (age, BMI) were introduced into the regression model, and the *P*-value for the interaction effect was calculated. In addition, restricted cubic spline curves and threshold effect analysis were employed to explore dose-response relationships between varying iodine nutritional statuses and blood lipid profiles. The tests were two-tailed, with statistical significance defined as *P* < 0.05.

## Results

### General characteristics of the study population

Table 1 lists the lifestyle and sociodemographic traits of research participants with varying iodine nutritional statuses (UICs and SICs). The participants ranged in age from 6 to 17 years, with a mean age of 11years. Among them, 894 (50.57%) were male, and 874 (49.43%) were female. Significant differences in age and sex distributions were observed across different UIC categories. Among the SIC categories, only the age distribution exhibited significant variation.

**Table 1:** Sociodemographic and health characteristics of the study participants

Variables	*N* (%)	UIC				SIC				
		< 100 µg/L	100–300 µg/L	≥ 300 µg/L	*P*^1^ Value	< 63.50 µg/L	63.50–72.62 µg/L	72.62–82.28 µg/L	≥ 82.28 µg/L	*P*^2^ Value
Total	1768	153(8.65)	1068(60.41)	547(30.94)		442 (25)	442 (25)	442 (25)	442(25)	
Sex										
Male	894(50.57)	64(42.38)	525(49.16)	305(55.76)	0.003	228(51.58)	218(49.32)	219(49.55)	229(51.81)	0.822
Female	874(49.43)	89(58.17)	543(50.84)	242(44.24)		214(48.42)	224(50.68)	223(50.45)	213(48.19)	
Age group (years)										
6–8	358(20.25)	26(16.99)	234(21.91)	98(17.92)	< 0.001	72(16.29)	78(17.65)	110 (24.89)	98 (22.17)	< 0.001
9–11	611(34.56)	58(37.91)	388(36.33)	165(30.16)		127(28.73)	155(35.07)	164 (37.10)	165 (37.33)	
12–14	625(35.35)	47(30.72)	346(32.40)	232(42.41)		186(42.08)	169(38.24)	137(31.00)	133(30.09)	
15–17	174(9.84)	22(14.38)	100(9.36)	52(9.51)		57(12.90)	40(9.05)	31(7.01)	46(10.41)	
BMI										
Underweight	103(5.83)	14(9.15)	57(5.34)	32(5.85)	0.260	23(5.20)	29(6.56)	20(4.52)	31(7.01)	0.814
Normal weight	974(55.09)	81(52.94)	603(56.46)	290(53.02)		237(53.62)	240(54.30)	248(56.11)	249(56.33)	
Overweight	275(15.55)	21(13.73)	155(14.51)	99(18.10)		76(17.19)	66(14.93)	70(15.84)	63(14.25)	
Obese	416(23.53)	37(24.18)	253(23.69)	126(23.03)		106(23.98)	107(24.21)	104(23.53)	99(22.40)	

*Lifestyle indicators*: *BMI *Body mass index, *SIC *serum iodine concentration, *UIC *urinary iodine concentration. Data values are reported as *n* (weighted percentage). Percentages may not sum precisely to 100% due to rounding

### Lipid levels of the study participants

Table 2 presents unadjusted lipid biomarker concentrations according to iodine nutritional status, age, sex, and BMI. The findings revealed statistically significant variations in TG levels among students with varying SICs (*P* < 0.001), as well as statistically significant differences in both TG and TC levels across different age groups, sexes, and BMI categories (*P* < 0.01). Specifically, compared to SIC (63.50–72.62 µg/L and 72.62–82.28 µg/L), TG levels were significantly elevated in both the SIC < 63.50 µg/L and SIC ≥ 82.28 µg/L groups. Female students presented higher TC and TG levels than male students did. TG levels gradually increased with age, whereas TC levels gradually decreased. Students with a normal BMI and underweight BMI had lower TC and TG levels than did those with overweight and obese BMIs.

**Table 2:** Unadjusted lipid levels, stratified by iodine intake, sex, age, and BMI of the study participants

		TG [mmol/L, M (P_25_, P_75_)]	TC [mmol/L, M (P_25_, P_75_)]	*P*^1^ Value	*P*^2^ Value
Iodine intake					
UIC	< 100 µg/L	0.87(0.72,1.17)	4.20(3.75,4.63)	0.124	0.311
	100–300 µg/L	0.83(0.65,1.11)	4.24(3.76,4.69)		
	≥ 300 µg/L	0.85(0.65,1.16)	4.33(3.71,4.81)		
SIC	< 63.50 µg/L	0.90(0.68,1.18)	4.25(3.75,4.70)	< 0.001	0.451
	63.50–72.62 µg/L	0.81(0.64,1.08)	4.24(3.72,4.69)		
	72.62–82.28 µg/L	0.80(0.63,1.06)	4.20(3.76,4.75)		
	≥ 82.28 µg/L	0.85(0.69,1.18)	4.29(3.81,4.77)		
Sex	Male	0.81(0.62,1.10)	4.19(3.70,4.70)	< 0.001	0.014
	Female	0.87(0.70,1.16)	4.28(3.82,4.75)		
Age	6–8 years	0.71(0.56,0.90)	4.38(3.97,4.89)	< 0.001	< 0.001
	9–11 years	0.82(0.63,1.12)	4.33(3.88,4.84)		
	12–14 years	0.91(0.71,1.20)	4.11(3.63,4.64)		
	15–17 years	0.96(0.78,1.26)	3.94(3.56,4.36)		
BMI	Underweight	0.77(0.60,1.04)	4.15(3.81,4.59)	< 0.001	0.019
	Normal weight	0.77(0.62,1.00)	4.20(3.72,4.65)		
	Overweight	0.90(0.70,1.18)	4.32(3.78,4.89)		
	Obese	1.00(0.78,1.46)	4.32(3.83,4.82)		

*BMI* Body Mass Index, *TC *Total cholesterol, *TG *Triglycerides, *P*^1^ and *P*^2^ values are based on the Kruskal-Wallis test

### Analysis of the relationships between the distribution and abnormality rates of different iodine indicators (UICs and SICs) and blood lipid levels

**Fig. 1:**
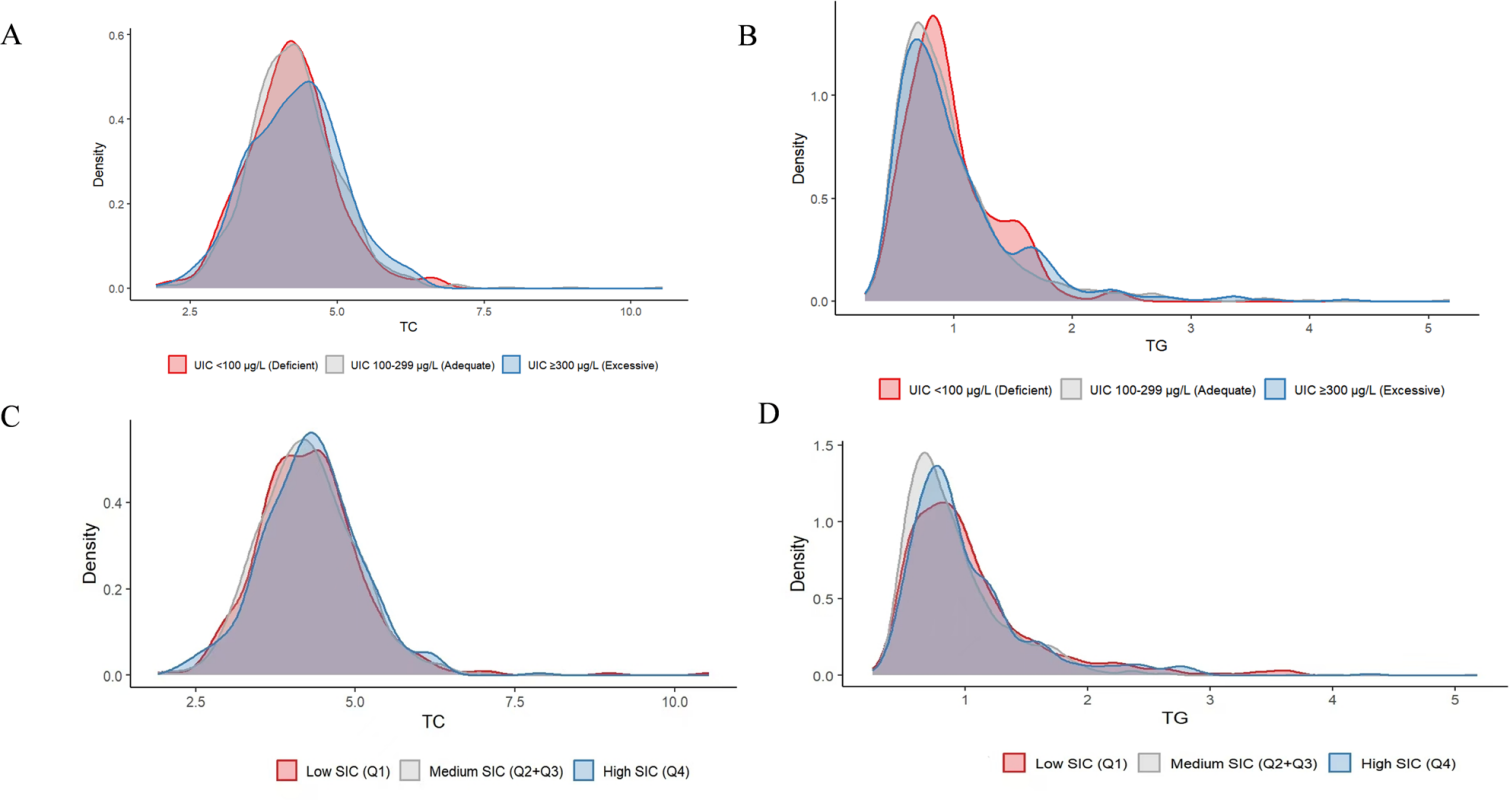
Lipid concentration distribution curves under different iodine nutritional statuses. Note: **A** and **B** represent the TC and TG concentration distribution curves under different UIC statuses; **C** and **D** represent the TC and TG concentration distribution curves under different SIC statuses

**Fig. 2:**
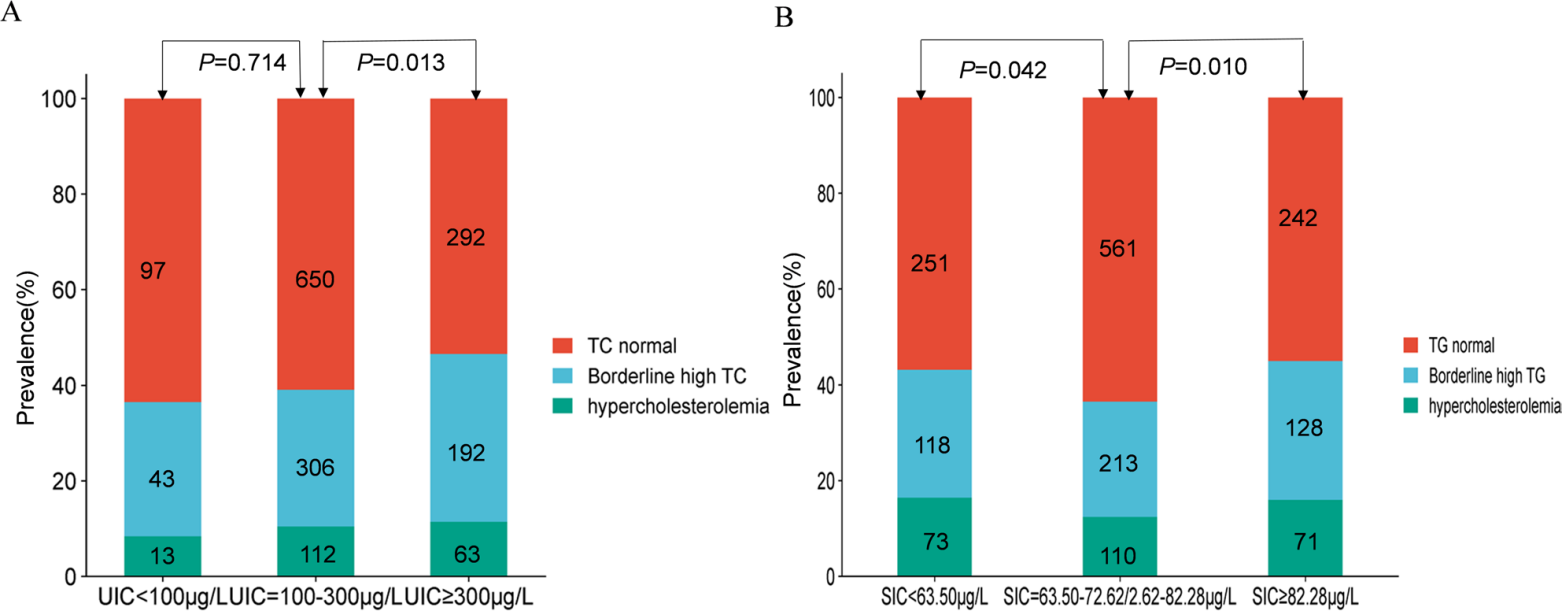
Relationships between the prevalence of dyslipidemia in children and different iodine nutritional statuses. Note: **A** represents the prevalence of TC and TG at different UIC levels; **B** represents the prevalence of TC and TG at different SIC levels

Figure 1 shows the kernel density distribution curves of blood lipid levels in populations with different iodine nutritional statuses. Each curve reflects the concentration trends and distribution characteristics of TC and TG in populations with varying UIC and SIC levels. As shown in Fig. 1A, the distribution curve for UICs ≥ 300 µg/L was the broadest and exhibited a pronounced right-tailing pattern. As shown in Fig. 1B, the distribution curve for UIC < 100 µg/L shifted to the right, with a more concentrated TG distribution. Figure 2A illustrates the TC levels across different UIC ranges. The chi-square test revealed statistically significant differences between serum TC levels and different UIC conditions (*P* = 0.034). Pairwise comparisons further revealed significant differences in hypercholesterolemia prevalence between the 100–300 µg/L UIC (10.49%) and the ≥ 300 µg/L UIC (11.52%) (*P* = 0.013).

The distribution patterns of the three groups in Fig. 1C are largely similar, with peaks occurring at TC values of 4.5. These findings indicate that the overall distribution trend of TC remains relatively consistent regardless of the serum iodine level. In Fig. 1D, the overall curves for SIC < 63.50 µg/L and SIC ≥ 82.28 µg/L both show a marked rightward shift and flatter profiles, suggesting higher average TG levels and greater intra-group variability. Notably, the curve for the high SIC group exhibited a pronounced long right tail, indicating a substantial proportion of individuals with exceptionally high triglyceride levels within this cohort. Figure 2B illustrates the prevalence of dyslipidemia across different SIC levels. The chi-square test indicated statistically significant variations in serum TG levels across different SIC conditions (*P* = 0.019). Pairwise comparisons revealed that the prevalence of hypertriglyceridemia in the < 63.50 µg/L (16.48%)and ≥ 82.28 µg/L SIC groups (16.10%)was significantly different from that in the 63.50–72.62 µg/L and 72.62–82.28 µg/L SIC (12.44%) (*P* = 0.039 and 0.011, respectively).

### Specific association between urinary iodine, serum iodine and dyslipidemia

Univariate logistic regression analysis of elevated TC revealed that after adjusting for age, gender, and BMI in the study subjects, the results indicated a significant positive correlation between UIC ≥ 300 µg/L and TC levels (*P* < 0.001), with an OR of 1.455 (95% CI: 1.175,1.802) (Table 3).

**Table 3:** Factors associated with elevated TC

Variables	Model 1		Model 2	
	cOR (95%CI)	*P*^1^ values	aOR (95%CI)	*P*^2^ values
UIC				
100–299 µg/L	1.00 (Reference)		1.00 (Reference)	
< 100 µg/L	0.898(0.632,1.275)	0.547	0.919(0.642,1.316)	0.646
≥ 300 µg/L	1.358(1.103,1.672)	0.004	1.455(1.175,1.802)	< 0.001
SIC				
63.50-72.62/72.62–82.28 µg/L	1.000 (Reference)		1.000 (Reference)	
< 63.50 µg/L	1.071(0.849,1.351)	0.563	1.168(0.920,1.482)	0.202
≥ 82.28 µg/L	1.237(0.982,1.558)	0.071	1.258(0.994,1.592)	0.056

*aOR* adjusted odds ratio (adjusted for age, sex, and body mass index), *CI *Confidence Interval, *cOR *crude odds ratio, *SIC *serum iodine concentration, *UIC *urinary iodine concentration

Both borderline high values and hypercholesterolemia, are collectively referred to as elevated TC. Using multivariate logistic regression analysis, the odds ratios for participants who developed elevated TC were estimated in two models: unadjusted (Model 1) and adjusted for sex, age, and BMI (Model 2). The *P*-values obtained from the multivariate logistic regression model in Model 2, with dyslipidemia diagnosis as the prognostic variable, are indicated

Univariate logistic regression analysis for elevated TG revealed that after adjustment in Model 2, abnormal serum TG levels showed significant positive correlations with SIC < 63.50 µg/L and SIC ≥ 82.28 µg/L (*P* = 0.033, *P* < 0.001), with OR values of 1.305 (95% CI: 1.022,1.665) and 1.536 (95% CI: 1.204,1.960), respectively (Table 4).

**Table 4:** Factors associated with elevated TG

Variables	Model1		Model2	
	cOR (95%CI)	*P*^1^ values	aOR (95%CI)	*P*^2^ values
UIC				
100–299 µg/L	1.000 (Reference)		1.000 (Reference)	
< 100 µg/L	1.180 (0.837,1.662)	0.345	1.122 (0.784,1.605)	0.529
≥ 300 µg/L	1.120 (0.908,1.381)	0.290	1.128 (0.906,1.406)	0.282
SIC				
63.50-72.62/72.62–82.28 µg/L	1.000 (Reference)		1.000 (Reference)	
< 63.50 µg/L	1.322(1.047,1.665)	0.019	1.305(1.022,1.665)	0.033
≥ 82.28 µg/L	1.422(1.128,1.794)	0.003	1.536(1.204,1.960)	< 0.001

*cOR *crude odds ratio, *SIC *serum iodine concentration, *UIC *urinary iodine concentration, *aOR *adjusted odds ratio (adjusted for age, sex, and body mass index), *CI *Confidence Interval

Both borderline high values and hypertriglyceridemia, are collectively referred to as elevated TG. Using multivariate logistic regression analysis, the odds ratios for subjects developing elevated TG were estimated in two models: unadjusted (Model 1) and adjusted for gender, age, and BMI (Model 2). The *p*-values obtained from the multivariate logistic regression model in Model 2, with elevated TG diagnosis as the prognostic variable

### Subgroup analysis and interaction analysis

**Fig. 3:**
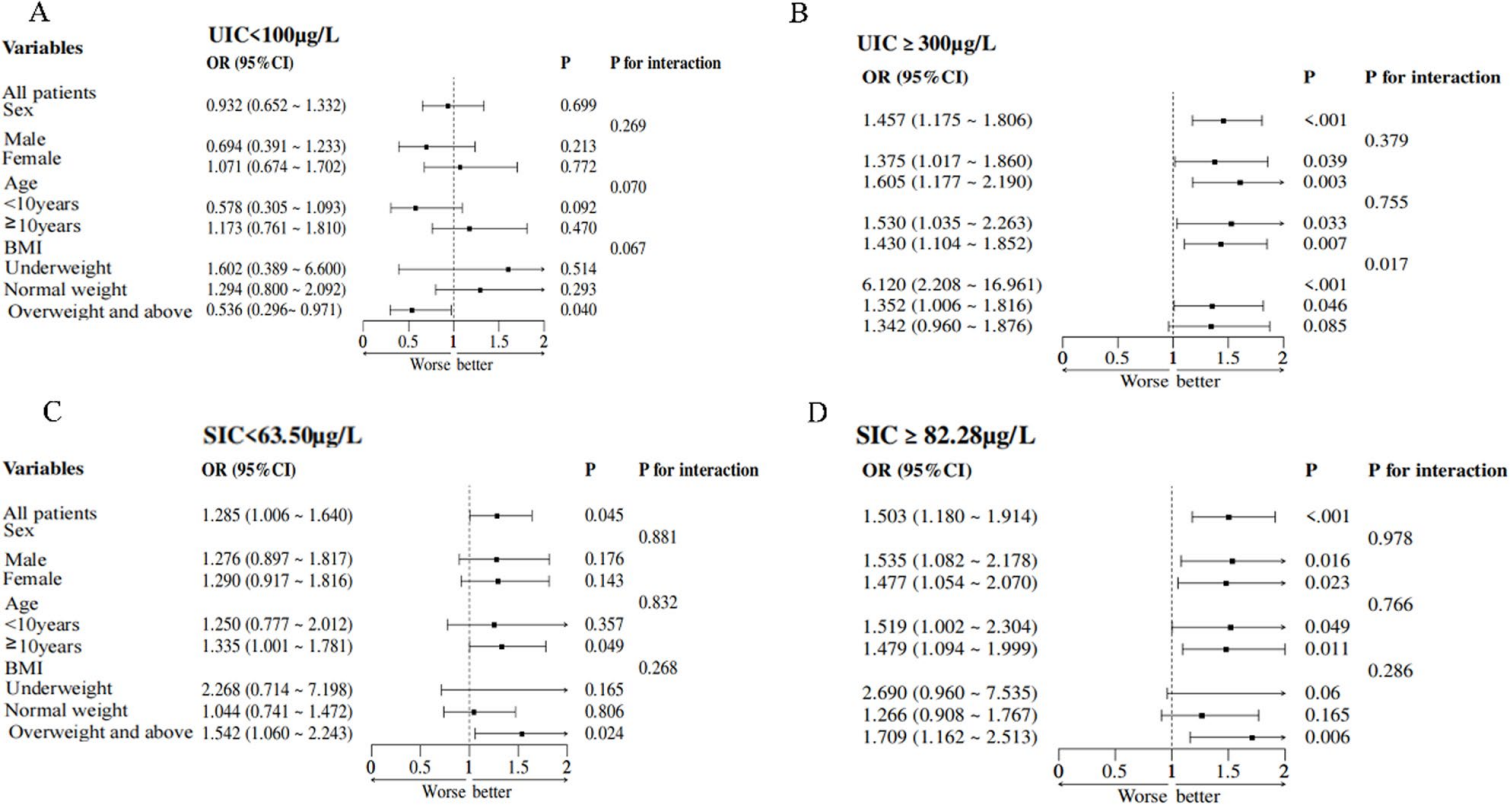
Effects of UIC and SIC on TC and TG levels in study subjects stratified by sex, age, and BMI. Note: Analyses adjusted for covariates including age, sex, and BMI. **A** and **B** are compared with UIC = 100–300 µg/L; **C** and **D** are compared with SIC = 63.50-72.62/72.62–82.28 µg/L; original significance level: α = 0.05, BMI stratified into 3 tiers, with 2 groups compared per tier, totaling 6 comparisons. Bonferroni corrected significance level: α’ = 0.05 / 6 ≈ 0.0083

Figures 3A and B illustrate the relationship between UIC and dyslipidemia across different pediatric and adolescent subgroups. Within BMI subgroups, compared to UIC = 100–300 µg/L, UIC ≥ 300 µg/L was significantly associated with serum TC abnormalities (*P* for interaction = 0.012). Continuous interaction analysis revealed a significant interaction between UIC and BMI (*P* for interaction = 0.025), indicating that the association of UIC with TC abnormalities is enhanced with increasing BMI. In contrast, the interaction between UIC and age did not reach statistical significance (*P* for interaction = 0.053) (Supplementary Table 4). After applying the Bonferroni correction, for BMI-underweight individuals, higher urinary iodine concentrations correlated with a greater likelihood of developing dyslipidemia (OR = 6.120, 95% CI: 2.208–16.961, *P* < 0.001). Furthermore, no significant interactions were observed for age or sex. Figure 3C and D illustrate the relationships between SIC levels and dyslipidemia across different subgroups. Although the association between an SIC ≥ 82.28 µg/L and TG abnormalities was more pronounced in overweight and obese individuals and those aged ≥ 10 years, no significant interaction was detected. The association between an SIC ≥ 82.28 µg/L and TG abnormalities was more pronounced in individuals who were overweight or had a higher BMI (*P* = 0.006 < 0.0083), although the interaction did not significantly differ.

### Dose-response relationship among UIC, SIC, and dyslipidemia

**Fig. 4:**
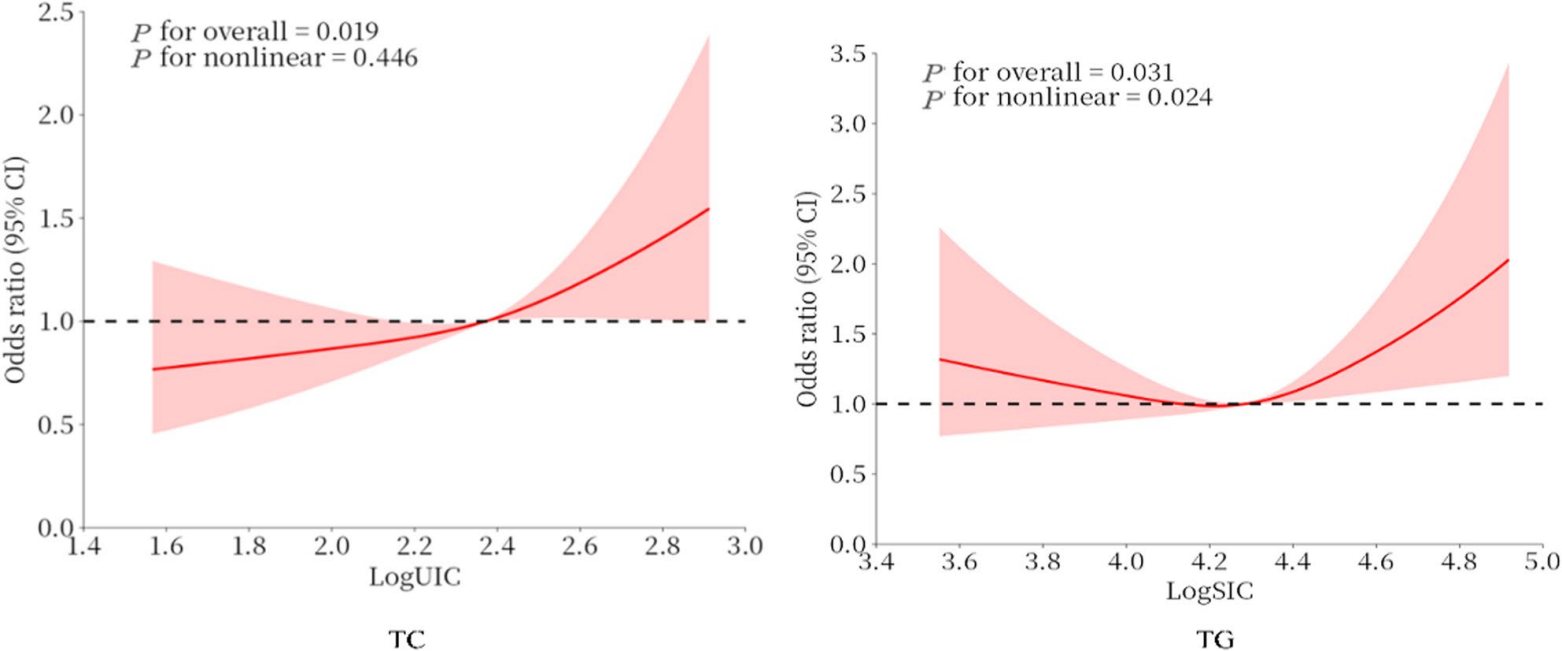
Restricted cubic spline analysis of the relationships between different iodine nutrition levels and blood lipids. Note: These lines represent adjusted odds ratios (solid lines) and 95% confidence intervals (long dashed lines) based on the restricted cubic spline model for log-transformed UIC and SIC levels. Nodes are set at the 10th, 50th, and 90th percentiles. Adjusted covariates include gender, age, and BMI

As illustrated in Fig. 4, restricted cubic spline (RCS) analysis revealed that UIC and SIC exhibited nonlinear dose-response relationships with TC and TG, respectively (*P* for nonlinear association = 0.019, *P* for overall association = 0.446; *P* for nonlinear association = 0.031, *P* for overall association = 0.024). Further threshold effect analysis indicated that SIC and TG exhibited threshold effects at 74.86 µg/L (*P* < 0.05).

### Relationships between thyroid hormone levels and blood lipid levels

To explore the connection between thyroid function and blood lipid levels more thoroughly, 397 respondents were randomly selected for thyroid hormone testing. The correlation analysis results (Table 5) revealed that, except for a significant negative correlation between Free Thyroxine (FT4) and TG (*P* < 0.001), notable links were observed between other thyroid function measures and lipid levels.

**Table 5:** Relationship between thyroid hormone levels and blood lipid levels

	TG		TC	
	*r*_s_	*P*	*r*_s_	*P*
FT_3_(pmol/L)	-0.051	0.311	-0.030	0.577
FT_4_(pmol/L)	-0.247	< 0.001	0.004	0.937
TSH (mIU/L)	0.031	0.534	-0.014	0.775

*r*_*s*_ represents *Spearman*’s rank correlation coefficient, which was used to test for linear correlations between thyroid hormone levels and lipid profiles, *FT3 *free triiodothyronine, *FT4 *free thyroxine, *TSH *thyroid-stimulating hormone

## Discussion

According to data from the World Health Organization (WHO), one-third of ischemic heart disease cases worldwide can be attributed to high cholesterol [19]. Research indicates that childhood is a critical period for preventing cardiovascular disease through primary interventions at the population level, making lipid levels in children a significant concern [8]. As effective indicators for assessing pediatric lipid profiles, changes in TC and TG levels warrant close monitoring. The relationship between iodine nutritional status and lipid levels is complex and contradictory. Domestic and international studies indicate that varying iodine intake may increase dyslipidemia and cardiovascular risk in adolescents and adults [20]. Thus, exploring the driving factors behind children’s blood lipid levels is of significant importance. This research methodically examined the link between iodine nutrition and blood lipid levels in children and teenagers, evaluating external environmental iodine intake through urinary iodine levels and internal environmental homeostasis through serum iodine levels.

Current evidence indicates that dyslipidemia is shaped predominantly by genetic predispositions, dietary patterns, and endocrine–metabolic factors, with dietary imbalance emerging as the most influential contributor [17, 18]. Overall, the findings demonstrate that girls had higher TC and TG levels than boys in this study, which is consistent with findings from a U.S. pediatric study [21]. These sex differences may be related to dietary patterns. Analysis of lipid levels across different age groups revealed that TC gradually decreases with age, whereas TG progressively increases. This pattern may be associated with hormonal and metabolic variations during childhood growth and development [22]. It should be noted that the subjects of this study are elementary and middle school students who are currently enrolled, indicating that school feeding programs may account for a significant proportion of iodine intake among school-aged children. Furthermore, UIC can serve as a comprehensive biomarker reflecting recent iodine intake. Therefore, this study incorporated only age, gender, and BMI as covariates in its adjustment, resulting in a relatively limited set of adjustments. Additionally, this study randomly selected a subset of participants for thyroid function testing. Owing to potential limitations in sample size, no significant correlations were observed between lipid levels and other thyroid indicators aside from FT4.

Iodine, an essential trace element, is associated with nutritional status and conditions such as goiter, hypothyroidism, and dyslipidemia [23]. FT4 and triglycerides were negatively correlated, whereas FT3 had a negative effect on total cholesterol under high urinary iodine exposure, suggesting that changes in thyroid hormones may influence lipid levels—a finding that is consistent with most studies [24]. This study revealed that children and adolescents with either high urinary iodine or high serum iodine levels presented a significantly elevated risk of dyslipidemia. From the perspective of urinary iodine, both kernel density plots and pairwise comparisons revealed differences in TC levels between the UIC ≥ 300 µg/L group and the UIC 100–300 µg/L group. This may be related to the larger sample size of the present study. Further logistic regression analysis revealed that after adjusting for all potential confounders, a UIC ≥ 300 µg/L was strongly linked to abnormal TC.

Existing animal studies and epidemiological investigations also indicate that long-term excessive iodine intake affects lipid metabolism [25, 26]. This study further examined how age, sex, and BMI stratification modify the association between varying levels of urinary iodine concentration and the risk of total cholesterol abnormalities. Analysis revealed that UICs ≥ 300 µg/L constitute a risk factor for abnormal TC in children, with heightened risk, particularly among underweight children. These findings indicate that underweight individuals might be more susceptible to the negative impact of high iodine levels on total cholesterol. By contrast, obesity appears to substantially confound the association between iodine exposure and circulating lipid profiles, complicating the interpretation of this relationship.

Iodine intake and blood lipids have been found to have a U-shaped association by both domestic and international research. Iodine deficiency increases the risk of hypercholesterolemia, whereas iodine supplementation improves insulin and lipid profiles in children [27, 28]. Several animal studies have confirmed that excess iodine can disrupt lipid metabolism in animals, resulting in a dose-response relationship with hyperlipidemia [29, 30]. Both high and low serum iodine concentrations are risk factors for aberrant serum triglyceride levels, according to this study’s analysis of serum iodine levels. Further regression-based classification and threshold effect analyses revealed a nonlinear dose-response relationship between SIC and TG, indicating a U-shaped curve between the serum iodine and triglyceride levels.

After adjusting for confounding factors, high iodine levels were significantly associated with increased dyslipidemia, indicating that while iodine is essential, excessive levels may be detrimental to lipid health. Interestingly, across all subgroups, high SIC consistently showed a significant association with triglyceride risk. These findings suggest that excess iodine may adversely affect triglyceride metabolism. Overweight/obesity emerged as a key effect modifier. The U-shaped relationship was most pronounced and statistically significant among overweight and obese children.

The total cholesterol in the blood that is present in different lipoproteins is referred to as TC. It is a crucial part of every cell membrane and can be used to determine a person’s risk of developing chronic cardiovascular disease [31]. This study discovered that high levels of TC are associated with increased urine iodine. This suggests that excessively high levels of iodine intake in the external environment may exert persistent, subtle effects on thyroid function, potentially inducing subclinical thyroid alterations. This, in turn, may suppress hepatic LDL receptor activity, slow LDL-C clearance, and lead to chronic, sustained increases in TC levels [16]. Triglycerides, as the principal form of stored metabolic energy, display considerable variability and offer a direct indicator of the body’s overall energy balance and metabolic state [31, 32]. This study identified a significant dose-response relationship between serum iodine concentrations and elevated triglycerides levels; however, no mediating role for thyroid hormones was observed. These findings suggest that iodine may exert effects independently of, or potentially synergistically with, thyroid hormones [33], and that the risk of long-term cardiovascular disease may be raised by both an excess and a lack of iodine. Beyond thyroid hormone-dependent pathways, fluctuations in serum iodine reaching certain thresholds may directly or indirectly trigger oxidative stress by activating neutrophils to produce reactive oxygen species (ROS) in extra-thyroidal tissues, such as the liver [34, 35]. This process may slow TG clearance rates, which might in turn contribute to TG accumulation in the bloodstream.

Iodine possesses multiple physiological effects independent of thyroid hormones, including antimicrobial, anti-inflammatory, and antiproliferative activities. At high concentrations, iodine can also exert pro-oxidant effects, which could be crucial for cardiovascular health in general. Urinary iodine reflects recent intake and excretion, while serum iodine indicates circulating iodine levels within the body. These two biomarkers may participate differently in metabolism processes, influencing cholesterol and triglycerides homeostasis through distinct mechanisms. Thus, maintaining balanced iodine nutrition is crucial for systemic metabolic health.

### Strengths and limitations

This study is the first to explore the association between population and individual iodine nutritional status and dyslipidemia (TG and TC) in children and adolescents from the perspectives of urinary iodine and serum iodine, based on dual indicators. This discovery provides fresh epidemiological understanding, highlighting the need for improved lipid evaluation in areas with either too high or too low iodine levels. However, this study utilized cross-sectional data, which cannot establish causality or observe temporal changes in iodine status and lipid levels. Additionally, no rigorous statistical correction was applied to multiple comparisons, potentially increasing the risk of Type I error. To address this, we conducted sensitivity analyses using the Bonferroni correction, which demonstrated that the core conclusions remained robust. Nevertheless, readers should exercise caution when interpreting subgroup analysis results. Finally, this study observed the risk of dyslipidemia from high iodine levels in both urinary and serum iodine measurements. However, the limited impact of not fully incorporating potential factors such as family income and dietary iodine intake, as well as the unweighted effect estimation in the association analysis, necessitate caution when extrapolating the results and further validation.

## Conclusion

In summary, this study found an upward trend between UIC and increased risk of TC abnormalities and a U-shaped link between SIC and increased risk of TG abnormalities. Excessive urinary iodine may exert adverse effects through hypercholesterolemia, while serum iodine abnormalities (both deficient and excessive) may exert adverse effects through hypertriglyceridemia. These findings suggest that while continuing to implement the iodized salt policy, a dual monitoring system for iodine nutrition and lipid profiles in children and adolescents should be established. Particular emphasis should be placed on enhanced monitoring in high-iodine areas and overweight/obese populations to achieve precise prevention and control of lipid metabolic health in children and adolescents.

## Supplementary Information

Supplementary Material 1.

## Data Availability

Data will be made available on request.
