# Supplementary material for: A study on the differences in urinary iodine and serum iodine in relation to children’s iodine nutrition and lipids
Source: Lipids Health Dis. 2026 Jun 4;25:176. doi: 10.1186/s12944-026-02982-7 (PMC13455225; doi:10.1186/s12944-026-02982-7)
Supplement: Supplementary file 1 [file 12944_2026_2982_MOESM1_ESM.docx]

**Supplementary Table 1** Effects of UIC and SIC on TC and TG Levels in Study Subjects Stratified by Gender, Age, and BMI

|  |  | UIC<100µg/L | UIC=100-299µg/L | UIC≥300µg/L | *P*^1^ | *P*^2^ |
| --- | --- | --- | --- | --- | --- | --- |
|  |  | AOR(95%CI) | Reference | AOR(95%CI) |  |  |
| Elevated TC | Male | 0.694(0.391,1.233) | 1.000 | 1.375(1.017,1.806) | 0.213 | 0.039 |
|  | Female | 1.071(0.674,1.702) | 1.000 | 1.605(1.177,2.190) | 0.722 | 0.003 |
|  | Age<10 years | 0.578(0.305,1.093) | 1.000 | 1.530(1.035,2.263) | 0.092 | 0.033 |
|  | Age≥10 years | 1.173(0.761,1.810) | 1.000 | 1.430(1.104,1.852) | 0.470 | 0.007 |
|  | Underweight | 1.602(0.389,6.600) | 1.000 | 6.120(2.208,16.961) | 0.514 | <0.001 |
|  | Normal weight | 1.294(0.800,2.092) | 1.000 | 1.352(1.006,1.816) | 0.293 | 0.046 |
|  | Overweight and above | 0.536(0.296,0.971) | 1.000 | 1.342(0.960,1.876) | 0.040 | 0.085 |

|  |  | SIC<63.50µg/L | 63.50-72.62/72.62-82.28µg/L | SIC≥82.28µg/L | *P*^1^ | *P*^2^ |
| --- | --- | --- | --- | --- | --- | --- |
|  |  | AOR(95%CI) | Reference | AOR(95%CI) |  |  |
| Elevated TG | Male | 1.276(0.897,1.817) | 1.000 | 1.535(1.082,2.178) | 0.176 | 0.016 |
|  | Female | 1.290(0.917,1.816) | 1.000 | 1.477(1.054,2.070) | 0.143 | 0.023 |
|  | Age<10 years | 1.250(0.777,2.012) | 1.000 | 1.519(1.002,2.304) | 0.357 | 0.049 |
|  | Age≥10 years | 1.335(1.001,1.781) | 1.000 | 1.479(1.094,1.999) | 0.049 | 0.011 |
|  | Underweight | 2.268(0.714,1.472) | 1.000 | 2.690(0.960,7.535) | 0.165 | 0.060 |
|  | Normal weight | 1.044(0.741,1.472) | 1.000 | 1.266(0.908,1.767) | 0.806 | 0.165 |
|  | Overweight and above | 1.542(1.060,2.243) | 1.000 | 1.709(1.162,2.513) | 0.024 | 0.006 |

**Supplementary Table 2** Blood lipid levels under different iodine indices**.**

| Variables | N(%) | UIC | | | SIC | | | | *P* | *P* |
| --- | --- | --- | --- | --- | --- | --- | --- | --- | --- | --- |
|  |  | <100µg/L | 100-300  µg/L | ≥300µg/L | <63.50µg/L | 63.50-72.62µg/L | 72.62-82.28µg/L | ≥82.28µg/L |  |  |
| TG |  | | | | | | | | | |
| normal | 1055(59.67) | 87(56.86) | 650(60.86) | 318(58.14) | 251(56.79) | 281(63.58) | 280(63.35) | 243(54.98) | 0.421 | 0.020 |
| borderline high | 459(25.96) | 42(27.45) | 278(26.03) | 139(25.41) | 118(26.70) | 98(22.17) | 115(26.02) | 128(28.96) |  |  |
| hypertriglyceri-demia | 254(14.37) | 24(15.69) | 140(13.11) | 90(16.45) | 73(16.51) | 63(14.25) | 47(10.63) | 71(16.06) |  |  |
| TC |  | | | | | | | | | |
| normal | 1039(58.77) | 97(63.40) | 650(60.86) | 292(53.38) | 260(58.82) | 265(59.95) | 269(60.86) | 245(55.43) | 0.034 | 0.642 |
| borderline high | 541(30.60) | 43(28.10) | 306(28.65) | 192(35.10) | 135(30.54) | 136(30.77) | 124(28.05) | 146(33.03) |  |  |
| hypercholestero-lemia | 188(10.63) | 13(8.50) | 112(10.49) | 63(11.52) | 47(10.63) | 41(9.28) | 49(11.09) | 51(11.54) |  |  |

**Supplementary Table 3** Relationship between SIC and TG after quartile merging

| Variables | Total  (n=1768) | SIC<63.50µg/L  (n = 442) | 63.50-72.62/72.62-82.28µg/L  (n = 884) | SIC≥82.28µg/L  (n = 442) | *χ²* | *P* |
| --- | --- | --- | --- | --- | --- | --- |
|  |  |  |  |  |  |  |
| TG, n(%) |  |  |  |  | 11.77 | 0.019 |
| normal | 1055(59.67) | 251 (56.79) | 561 (63.46) | 243 (54.98) |  |  |
| borderline high | 459 (25.96) | 118 (26.70) | 213 (24.10) | 128 (28.96) |  |  |
| hypertriglyceridemia | 254 (14.37) | 73 (16.52) | 110 (12.44) | 71 (16.06) |  |  |

**Supplementary Table 4** Continuous Interaction analysis

| Elevated TG | Variables | Model1 | | Model2 | |
| --- | --- | --- | --- | --- | --- |
|  |  | cOR (95%CI) | *P*^1^ values | aOR (95%CI) | *P*^2^ values |
|  | SIC*BMI | 1.001 (1.001 ~ 1.001) | <0.001 | 1.001 (1.001 ~ 1.001) | 0.049 |
|  | SIC*Age | 1.000 (1.000 ~ 1.001) | 0.055 | 1.000 (1.000 ~ 1.001) | 0.113 |
|  | UIC*BMI | 1.001 (1.001 ~ 1.001) | <0.001 | 1.000 (1.000 ~ 1.000) | 0.125 |
|  | UIC*Age | 1.000 (1.000 ~ 1.000) | 0.217 | 1.000 (1.000 ~ 1.000) | 0.264 |
| cOR: crude odds ratio, SIC: serum iodine concentration. UIC: urinary iodine concentration. aOR: adjusted odds ratio (adjusted for age, sex, and body mass index) CI: Confidence Interval. | | | | | |

| Elevated TC | Variables | Model1 | | Model2 | |
| --- | --- | --- | --- | --- | --- |
|  |  | cOR (95%CI) | *P*^1^ values | aOR (95%CI) | *P*^2^ values |
|  | SIC*BMI | 1.000 (1.000 ~ 1.000) | 0.393 | 1.000 (1.000 ~ 1.000) | 0.674 |
|  | SIC*Age | 0.999 (0.999 ~ 0.999) | <0.001 | 1.000 (1.000 ~ 1.001) | 0.587 |
|  | UIC*BMI | 1.000 (1.000 ~ 1.000) | 0.121 | 1.001 (1.001 ~ 1.001) | 0.025 |
|  | UIC*Age | 1.000 (1.000 ~ 1.000) | 0.283 | 1.000 (1.000 ~ 1.000) | 0.053 |
| cOR: crude odds ratio, SIC: serum iodine concentration. UIC: urinary iodine concentration. aOR: adjusted odds ratio (adjusted for age, sex, and body mass index) CI: Confidence Interval. | | | | | |

**Supplementary Figure 1** Restricted cubic spline analysis of the relationships between different iodine nutrition levels and blood lipids

**Supplementary Figure 2** Sampling frame
